# Semi-professional language mediators in patient-provider interactions in Germany: an interview study

**DOI:** 10.1186/s12913-025-12832-4

**Published:** 2025-05-09

**Authors:** Hanna Luetke Lanfer, Victoria Touzel, Doreen Reifegerste

**Affiliations:** School of Public Health, Bielefeld University, Universitaetsstrasse 25, Bielefeld, 33615 Germany

**Keywords:** Language mediation, Healthcare interpreting, Triadic communication, Qualitative methods

## Abstract

**Background:**

In multilingual healthcare settings, language mediators play a critical role in facilitating communication between patients and providers who do not share a common language. Existing research has developed theoretical role typologies for professional interpreters– such as conduit, clarifier, cultural broker, and advocate. It remains unclear how these roles apply to semi-professional language mediators (i.e., individuals with some training but no formal certification), increasingly relied upon in Germany. The lack of institutional oversight, combined with culturally diverse patient-provider dynamics, introduces role ambiguity and potential ethical conflicts. This study investigates how semi-professional language mediators define their roles and navigate role-related conflicts within healthcare encounters.

**Methods:**

We conducted qualitative interviews with 25 participants (10 language mediators and 15 physicians who regularly act as language mediators), reflecting a range of linguistic, cultural, and professional backgrounds. Interviews focused on competencies, experiences, and role perceptions. Data were collected in German, transcribed, and analyzed using qualitative content analysis, guided by an established interpreter role typology.

**Results:**

The four role types—conduit, clarifier, cultural broker, and advocate—were all identified in the data. However, participants described their work as highly fluid, shifting between roles depending on situational demands. While neutrality (conduit) was emphasized as institutionally desirable, mediators frequently intervened to clarify, adapt, or advocate based on patients’ needs and cultural expectations. This balancing act created tensions between professional boundaries and ethical obligations, especially in emotionally charged or culturally complex situations.

**Discussion:**

The findings suggest the need for clearer role awareness and targeted training to prepare semi-professional language mediators for the complexities they encounter in healthcare settings. There is a need for targeted, certified training that goes beyond language skills to include cultural competence, emotional communication, and ethical considerations, including the awareness of different roles. Addressing these training needs is essential to improving the quality of care for linguistically and culturally diverse populations.

**Supplementary Information:**

The online version contains supplementary material available at 10.1186/s12913-025-12832-4.

## Introduction

Due to global migration and refugee movements, many Western societies are becoming increasingly linguistically and culturally diverse [1–3]. In Germany, around a quarter of the population has a so-called migration background, defined as either directly migrated to Germany, or with at least one parent who was not born in Germany [4]. Since 2013, immigration movements to Germany have changed compared to previous decades, with an increasing number of people from countries in the Middle East and, most recently, Ukraine [5, 6]. Despite state-facilitated language and integration courses, communication difficulties persist between migrants and healthcare providers, exacerbated by cultural and systemic differences, especially following arrival [7]. These challenges are particularly pronounced in sectors like healthcare, where it has been shown that the discordance of a common language in doctor-patient-interactions can lead to misdiagnosis [8], longer hospital stays [9], non-compliance with treatments [10], unnecessary hospital visits [11] and patient dissatisfaction [12].

In the absence of a shared language between healthcare provider and patient, a third person^1^ – a language mediator – can be employed to facilitate understanding by transmitting written or verbal communication into the other language on an ad-hoc basis [13–15]. While language mediators can help both patients and providers to convey health issues, treatment plans, and health information more precisely and foster shared decision-making [16], these encounters shift the traditional dyadic doctor-patient interaction into a triadic one [17] and thereby alter the communication situation [16, 18]. In direct doctor-patient exchanges, meaning is negotiated between two interlocutors while in mediated interactions, a third party, namely the language mediator, becomes an integral part of the exchange. This transformation introduces complexities, as information no longer moves directly between speaker and listener but is relayed through an intermediary. Two reviews of language mediator impact on clinical outcomes and patients’ satisfaction show that language mediation can improve communication quality, patient satisfaction, and clinical outcomes, but it also carries risks such as omissions and unintended influence on the flow or content of medical interactions, particularly when interpreters are not adequately trained [19, 20]. The authors highlight the importance of interpreter roles and responsibilities in healthcare language mediation to ensure ethical boundaries and effective communication for both patients and providers. The need for role definitions has been widely acknowledged in professional interpreting, where research has established role typologies. Distinctions between different roles like conduits, clarifiers, cultural brokers, or advocates [21–23] guide professional training and interpreters in their interactions. However, studies show that those who translate and interpret healthcare interactions in Germany come from a variety of different backgrounds [17]. These range from professionally trained interpreters *(i.e.*,* individuals with several years of formal education and certification*,* such as a university program)*, semi-professionals *(i.e.*,* individuals with some training*,* like short courses)*, to untrained individuals like family members *(i.e.*,* those with no formal training who assist based on language familiarity)* [24, 25]. Given the changes in immigration flows in Germany over the past decade [4], there is a shortage of trained translators and interpreters for languages that were previously in low demand, such as Dari, Pashto, Kurdish, and their dialects, making semi-professionals an increasingly central part of healthcare services [17]. While role typologies have been applied to professional interpreters in both studies and training [16], their relevance to semi-professional mediators remains unclear in the German context, especially as they often operate with varying degrees of training, and lack of institutional oversight. To examine how semi-professional language mediators conceptualize their own roles, this study asks:

- RQ 1: How do semi-professional language mediators define their role(s) for language mediation in healthcare encounters?

The concept of language mediation refers not only to converting words from one language to another, but also includes the transmission of meanings, cultural nuances, and contextual information [21, 26, 27]. The International Medical Interpreters Association [28] emphasizes that language mediators are not neutral communication channels but need to engage both provider and patient beyond the literal transmission of words to ensure critical socio-cultural assumptions embedded in communication are understood. In healthcare, this requires situational decisions about balancing linguistic accuracy with the need to adapt messages to different cultural and medical frameworks [27–33]. This implies that language mediators intervene in healthcare interactions which exposes them to navigating between multiple roles, thereby creating potential for role conflicts and ambiguity [20, 31, 34]. This raises a second question that this study seeks to respond to:

- RQ 2: How do semi-professional language mediators navigate (potential) conflicts within and between their roles in healthcare encounters?

## Methods

### Study design

This study employed qualitative interviews to explore the perspectives of semi-professional language mediators from different professional and socio-demographic backgrounds. The study focused on language mediation in general healthcare encounters, i.e., primary care, outpatient and hospital-based consultations, with a focus on languages such as Arabic, Farsi, Dari, Kurdish, and Pashto which reflect demographic shifts and current gaps in professional interpreter availability in the German healthcare system. This study is reported in accordance with the COREQ (Consolidated Criteria for Reporting Qualitative Research) checklist [35].

### Participants and recruitment

Given the wide range of individuals involved in language mediation, we focused on a specific subset within this group. On a spectrum ranging from professional language mediators with formal training in interpretation to relatives with no professional background and only occasional experience, we targeted individuals who interpret in healthcare encounters with some degree of training. Participants were recruited based on their experience with language mediation, not their primary profession. This includes those who work primarily for agencies or volunteer organizations offering language mediation services, e.g., non-profit and city-council-supported initiatives. Informed by our professional networks, we contacted five such organizations with invitations to suggest potential participants with language skills from Middle Eastern countries. Moreover, we also included bi- or multilingual physicians with training in language mediation who interpret for other physicians in hospital settings in addition to being a healthcare provider. A database maintained by the Association for Statutory Health Insurance Physicians (ASHIP), listing physicians who offer language mediation, served as a secondary recruitment source. Physicians were selected based on their proficiency in Middle Eastern languages, ensuring relevance to the study’s focus. Recruitment took place via email invitations sent to physicians directly. To ensure a diverse sample, recruitment was iterative. Initially, participants were included based on availability, resulting in a predominance of Arabic and Persian/Farsi-speaking mediators. As the study progressed, targeted efforts were made to include speakers of Dari, Pashto, Turkish, and Kurdish.

The sample included *n* = 10 language mediators and *n* = 15 physicians who regularly provided language mediation in addition to their medical duties. While the physicians had dual perspectives as both healthcare providers and mediators, their role as mediators was the primary focus of analysis. All participants had migration experience from a Middle Eastern country, reflecting the demographic shifts in Germany’s migration patterns in the past decade.

### Data collection

Interviews were conducted between November 2023 and January 2024 via Zoom video conferencing to enable nationwide participation. Each participant received an information sheet with detailed information on the interview procedure, data processing and data protection. After receiving the signed consent form, an appointment for the interview was arranged and conducted by HLL and VT, a postdoctoral researcher and a PhD student, both with extensive experience in qualitative research. The interview questions focused on four topics: (1) socio-demographic data and professional experiences of the participants; (2) characterization of migrant groups served by the participants; (3) medical consultations with language mediation, including roles, competencies, conflicts and the triadic relationship between doctor, patient and language mediator; and (4) experiences in roles as both migrant and language mediator in the German healthcare system (see supplementary material for the interview guide). The interviewees were not introduced to the role typologies, instead, participants were asked open-ended questions about their roles, key skills, and competencies required for language mediation. Upon mentioning terms such as neutrality or anonymity, they were prompted to elaborate with examples. Interviews lasted approximately 57 min, ranging from 32 to 81 min. An audio recording was made of each interview, which was verbatim transcribed with AI support (f4x audiotranskription, dr. dresing & pehl GmbH). The accuracy of the transcription was checked by a student assistant, and any personal data of the participants was pseudonymized. The audio recordings were then deleted. Data was collected in German, and quotes included in this manuscript were translated using AI-assisted tools and subsequently reviewed for accuracy.

### Analytical framework

To systematically analyze how semi-professional language mediators define their roles (RQ 1) and navigate role conflicts (RQ 2), this study applied qualitative content analysis (QCA) [36]. The analysis was guided by an established role typology developed in interpreting research, which distinguishes between four primary roles [21–23, 37–41].

- The conduit role involves the mediator transmitting messages as accurately as possible without adding or altering meaning. While often perceived as a neutral, invisible function, research highlights that complete neutrality is difficult to achieve in practice, as mediators must still decide how to structure statements and handle ambiguities [39, 42].
- The clarifier role goes beyond direct translation by addressing misunderstandings and explaining unfamiliar medical concepts. Mediators in this role assess when additional clarification is necessary, balancing accuracy with the need for comprehension [39].
- The cultural broker role involves interpreting not only linguistic content but also culturally embedded meanings, norms, and expectations that might otherwise lead to miscommunication. This role helps bridge intercultural gaps in healthcare encounters and can sometimes contribute to conflict resolution [23, 37, 43].
- The advocate role entails supporting the patient’s interests, sometimes intervening to ensure equitable treatment. While advocacy can be essential in certain contexts, it also raises ethical questions regarding impartiality [37, 38, 44].

To examine how semi-professional language mediators define their roles (RQ1), the analysis focused on identifying role attributes within the established typology of conduit, clarifier, cultural broker, and advocate. These attributes were inductively derived from the data, reflecting how mediators articulated their responsibilities in practice. To analyze how conflicts emerged within and between these roles (RQ2), the analysis adopted a spectrum approach to capture how mediators fluidly transition between different roles depending on the specific healthcare encounter. Rather than assigning rigid classifications, coding focused on identifying moments of role shifting, particularly between neutrality (conduit) and active engagement (advocate).

The coding framework was initially structured around the predefined role typologies of conduit, clarifier, cultural broker, and advocate. During the analysis of the first five interview transcripts, new subcategories emerged inductively based on role attributes identified in the data. These subcategories were iteratively refined to ensure distinct categories. The other transcripts were coded using this coding framework. New subcategories were only created if text segments did not match the previously created ones. Each transcript was coded by two coders (HLL and VT). At intervals of five transcripts each, differences in the coding were discussed and adjusted, which led to a further specification of the coding frame. MAXQDA software (VERBI GmbH) was used to support the computer-assisted data analysis.

To improve the clarity and coherence of the manuscript, AI-assisted tools (e.g., ChatGPT by OpenAI and DeepL) were employed for language refinement and translation of quotes.

## Results

### Sample demographics

A total of 25 individuals participated in the study. The surveyed group of language mediators (*n* = 10) consisted of those with a professional (referred to as PLM, *n* = 4) and those with a non-professional (NPLM, *n* = 6) healthcare background (i.e., individuals without formal education or training in healthcare-related fields such as medicine, nursing, or pharmacy) as well as bi- or multilingual physicians with language mediation experience (HCP, *n* = 15) exhibited diversity in terms of migration experience, cultural backgrounds and language skills (see Table 1 below). People from the first generation of immigrants dominated in the sample (*n* = 20 with direct migration experience). In terms of specialization, the majority of participants (*n* = 19) had participated in some training for language mediation, those without training were all physicians and had acquired experience ‘on the job’. Trainings varied greatly, ranging from a one-time three-hour course to a six-month certification program with examinations. We did not distinguish participants by training backgrounds because the diverse nature of their training made it impossible to accurately compare their acquired skills and competencies.

**Table 1:** Professional and sociodemographic data of recruited sample (*N* = 25)

Characteristics	n	%
Gender		
Female (F)	15	60
Male (M)	10	40
Participant background		
Physician with language mediation experience (HCP)	15	60
Language mediator without professional healthcare background (NPLM)	6	24
Language mediator with professional healthcare background (PLM)	4	16
Migration experience		
First generation	20	80
Second generation	5	20
Language skills (more than one possible)*		
Arabic	14	56
Persian/Farsi	7	28
Kurdish/Kurmanji	7	28
Turkish	6	24
Dari	4	16
Hebrew	1	4
Pashto	1	4
Azerbaijani	1	4
Aramaic	1	4
Tajik	1	4
Number of languages known		
1	14	56
2	4	16
3	7	28

*As our research focuses on language skills specific to languages spoken in the Middle Eastern region, skills in German or English are not included in this table. All participants spoke German

To distinguish participants from the different subsamples, the following terminology will be applied: ‘Participants’ includes perspectives from all subsamples. ‘Language mediators’ refers to those interviewees who primarily work in language mediation with (PLM) and without professional (NPLM) backgrounds in healthcare; ‘healthcare professionals’ (HCP) refers to the sample of physicians who primarily work as doctors, have training in language mediation and work as language mediators upon request. In addition, participants’ gender (F and M; none identified as non-binary) and country with whom the participants have familial or cultural connections are mentioned after sample quotes. While we use ‘language mediation’ throughout this manuscript as described earlier, some of our interviewees labelled their services as ‘interpreting’ whereas others used the term ‘language mediation’ – we adhered to their wording in quotations.

### Role definitions and attributes for semi-professional Language mediators in healthcare encounters (RQ1)

Overall, our analysis confirmed the presence of the four language mediator roles in healthcare encounters while also identifying role attributes that shape how semi-professional mediators define their responsibilities (see Table 2 and supplementary materials).

**Table 2:** Language mediator roles and identified attributes

Language mediator role	Role-specific attributes	Definition of attribute
Conduit	Neutrality	Conveying messages without adding personal opinions, emotions, or altering the content to maintain impartiality.
	Confidentiality	Ensuring that all information shared during the encounter remains private and is not disclosed beyond the interaction.
	Anonymity	Maintaining an impersonal presence, avoiding personal relationships with either party.
	Accuracy	Ensuring precise and faithful transmission of medical information, minimizing omissions and additions.
Clarifier	Clarifying terminology	Identifying and explaining/paraphrasing complex medical terms when necessary to prevent misunderstandings.
	Managing linguistic differences	Identifying and addressing challenges arising from dialects, accents, or colloquial expressions that may hinder mutual understanding.
	Communication facilitation	Adapting the structure or formulation of messages to support comprehension, e.g., rephrasing or simplifying.
	Managing the flow of doctor-patient-conversations	Coordinating the triadic encounter, including managing turn-taking, pace and length of speech, and when to summarize points.
Cultural broker	Cultural mediation	Bridging cultural differences by interpreting norms, behaviors, and meanings to enhance mutual understanding.
	‘Packing’ bad news	Conveying difficult medical information in a culturally sensitive way to align with patient expectations and emotional needs.
	Relaying decision-making norms	Clarifying differences in medical decision-making processes to help patients engage in doctor-patient discussions
	Providing additional explanations	Offering supplementary context to ensure patients understand medical procedures, expectations, and healthcare system norms.
Advocate	Ensuring proper medical practice	Intervening to prevent miscommunication or medical decisions that could negatively impact patient safety.
	Actively managing the interaction	Taking on a leadership role in the triadic encounter, e.g., by deciding when to intervene, reframe, or readdress points.
	Continuing support beyond the interaction	Assisting patients in navigating healthcare logistics, such as booking follow-ups or understanding prescriptions.

#### ***Conduit***

In our study, the role of the *conduit* – where the language mediator acts as a neutral channel – was consistently highlighted as crucial to language mediation in healthcare encounters. Every interview mentioned at least one characteristic associated with the conduit’s role, including neutrality, confidentiality, anonymity, and accuracy, prioritising effective and impartial communication between patients and healthcare providers. 

*Neutrality* emerged as a key characteristic for language mediators in their role as conduits. One physician highlighted this by saying:

“The interpreter is supposed to interpret, to convey what is being said. He should not bring in any emotions, he should not leave anything out. He should simply say what is now being said by the patient and also by the doctor.” (PLM M Syria)

*Confidentiality* was another frequently mentioned characteristic. Participants stressed the importance of trust built through confidentiality agreements:

“She [the interpreter] signs a confidentiality agreement, and then I say from my side, you can trust me. I have signed that I have confidentiality. … What you are telling me now will stay exactly here and will not end up anywhere else.” (NPLM F Iran)

*Anonymity* in terms of ensuring impartiality was articulated by multiple participants:

“We are anonymous, and they don’t know who we are. We don’t get a name, and they don’t get a name from us either. We are a neutral, anonymous person.” (NPLM F Iran)

*Accuracy* was also deemed a key characteristic, with mediators recognizing the potential consequences of inaccuracies in their interpretations:

“Generally, without an interpreter, there would be no communication at all because they really don’t understand a word. And medical topics are not subjects that can be conveyed with pictures or gestures. Therefore, it is really very important that everything is said precisely so that things can be communicated well.” (NPLM M Iran)

Handling different accents was described to be particularly challenging for being accurate, where participants described a need to be particularly attentive:

“A challenge for me is always the accent. If they have a strong accent, especially an Afghan accent. By now, I can understand 95 to 97% of it but sometimes there are words I don’t know. … But what I mean is also with doctors sometimes, they have a very, very strong accent. I feel that there are many foreign doctors with such a strong accent that sometimes they are really hard to understand.” (NPLM W Iran)

#### Clarifier

A need for the role of the *clarifier* – focused on ensuring linguistic clarity and resolving ambiguities in the conversation – was also frequently expressed by all participants. Especially language mediators without a professional background in healthcare were aware of *clarifying terminology* as potential sources of misunderstanding, seeking clarification when in doubt. Moreover, being adept at *managing linguistic differences* to ensure clear communication and mutual understanding was also described by participants. This includes paying attention as to what the patient might not understand due to accents or dialects, using simplifying language and providing examples to bridge gaps:

“You have to know that the Arabic language is one of the most difficult languages in the world, and there are many dialects, and people speak colloquially. There are also many cultural words that not every Arab will understand unless they come from the same culture. And that means for them that these patients actually don’t understand the medical language despite a shared language on paper. You have to bring it closer to them with very simple words, with many examples.” (HCP M Palestine).

*Facilitating communication* was considered a crucial trait as participants remarked that they were often more aware of which concepts needed to be broken down for a patient than the doctor. A series of follow-up questions or lengthy explanations, however, could cause tension, requiring careful communication between all parties:

“A very, very common question that arises in many appointments is: ‘Are you allergic to anything?’ Especially Afghan adolescents have difficulties with this question as many of them come from small villages in Afghanistan and don’t even know what an allergy means. So, I first have to explain, if you eat something, you have a negative reaction to it, and so on. So, I have to clarify the terms precisely, and they still don’t understand, and then it drags on. Then the doctor thinks, what’s going on here? Am I manipulating the conversation? I have to explain: ‘No, I first have to explain what an allergy means, what the consequences are, what the symptoms are, and so on, because the person has never experienced it.’” (NPLM M Iran).

*Managing the flow of doctor-patient-conversations* was often mentioned by the non-physician language mediators. For instance, dealing with longer explanations from doctors requires asking them to use shorter sentences so all parties understand crucial points. Moreover, balancing the conversation was described as complex when both parties have multiple questions and limited time, as language mediators needed to organize and prioritize information as well as interpreting. 

#### Cultural broker

Characteristics associated with the role of a cultural broker – bridging cultural gaps and ensuring that communication is not only linguistically accurate but also culturally sensitive – were mentioned the most in all interviews. It was notable that participants mostly described how they adapted their communication to the patients’ needs rather than the physicians’.

*Cultural mediation* was described as an important aspect of the cultural broker role. Participants emphasized the importance of knowing both cultures to effectively mediate and foster a better relationship between the patient and the healthcare provider, e.g., by providing cultural knowledge for both parties involved in the interaction. Other participants underscored the importance of emotional sensitivity in cultural mediation, particularly in how relationships are managed by immigrants. They explained that understanding the cultural emphasis on emotional connections helped improve the relationship between the patient and the doctor. According to participants, cultural mediation also includes conveying empathy, understanding, and reassurance to the patient. As such, language mediators considered their role as also creating a safe and trusted environment for patients, which helped them feel more comfortable and supported during medical interactions.

“It is a support for many to know that there is someone who understands me, who can relate to my culture. Regardless of whether it really leads to a groundbreaking solution or something happens with this feeling. Just having this familiarity, I believe, gives many the energy and courage to try again.” (PLM F Iran).

*‘Packaging’ bad news* was identified as a critical aspect of the cultural broker role. Participants explained that delivering difficult news, particularly about severe illness or death, required a sensitive and culturally appropriate approach. In cultures such as found in Middle Eastern countries, bad news is conveyed gently, with hope and reassurance, while this is communicated more directly in Germany. Mediators often found themselves having to balance these differing approaches to ensure that the patient could cope with the information:

“The hardest thing I have experienced in my 15-month career as an interpreter was having to tell a mother that her child would not survive much longer. … I knew how our culture works, and just transporting that in this situation, which was also a shock moment for me, was not easy. So that is my job, to somehow convey such situations in a way that the person can handle the bad news better, not freak out, not collapse, or something like that.” (NPLM M Iran).

*Relaying decision-making norms* was another often-mentioned function of the cultural broker role. Participants explained that the decision-making process in medical settings often varied greatly between cultures, with some cultures accustomed to approaches where doctors make decisions, while others emphasize shared decision-making. Participants described the importance of bridging this gap by explaining these differences to patients and ensuring they understood their role in the decision-making process, thereby reducing uncertainty:

“In Syria, the doctor decides. He says, ‘we do it this way’ and that’s it. He rarely leaves the decision to the patients. And here it is a bit difficult because here the doctor involves the patients and they make decisions together. For people from the Middle East, that is rather unusual. I’m not suggesting that this should change, but I think it needs a bit more explanation. As a doctor myself, I can add ‘I know this method is good, but I want you to know what you have. For me, it is important that you as a patient understand the treatments and you also make the decision. Not that I don’t know. I know exactly what is important.’”(HCP M Syria).

*Providing additional explanations* to ensure patients understood medical instructions and their significance was deemed a crucial aspect of the cultural broker role. Participants explained that often, patients were given medical documents or instructions that they did not fully understand. While they were aware of the knowledge gap, the German doctor often was not. In such cases, participants explained how they stepped in either during the interaction or afterward to clarify instructions in a way that the patient could comprehend.

#### Advocate

The advocate role, the most active role a language mediator can perform, was least often addressed, with instances found only among language mediators, not doctors.

*Ensuring proper medical practice* was described by participants in terms of how they used their medical knowledge and cultural understanding to advocate for practices that are safe and appropriate. For example, one participant recounted a situation where a paediatrician, unable to read a Syrian vaccination card, decided to re-vaccinate a child completely. The mediator, actively intervened and addressed the physician independently:

“I don’t just see myself as a language mediator; I cannot stand idly by when no information is being communicated to the patient or the doctor. Recently, I had a situation where a mother came to the paediatrician with a Syrian vaccination card, which was in Arabic. The paediatrician said, ‘I can’t do anything with this vaccination card, I will just vaccinate this child completely again.’ I know you don’t do that. I have a medical background, and vaccinations are not something to be done casually. I first informed the mother about this. She then asked me, ‘Why does he want to vaccinate my son completely again? My son is fully vaccinated.’ Then I translated this and asked the doctor, stepping out of my role as a language mediator and into my role as a medical professional, ‘What about checking the [antibody] titer?’ This example was my boundary because not saying ‘Stop’ would have been negligence on my part as a mediator; it would be bodily harm to re-vaccinate people completely again.” (PLM W Palestine).

*Actively managing the interaction* was a key aspect for language mediators, who were acutely aware of the triadic setting in which they operated. Communication flowed through them, giving them the ability to take an active role in managing the interaction. Participants described how they had to ensure that patients fully understood medical advice and instructions, sometimes repeating or rephrasing information to make sure nothing was missed. Participants noted cultural differences in communication styles and how they navigated these to facilitate understanding. For example, one mediator recounted how people Middle Eastern cultures rarely say ‘no’ due to cultural perceptions of politeness, which required a more active role in the interaction.

Participants described how they went beyond their formal duties, *continuing support beyond the interaction*, such as helping them navigate the healthcare system and access necessary services, offering ongoing assistance to ensure the patient’s well-being:

“Often, patients ask me if I can recommend someone or what I would do. Then I try to connect them with contacts, and it feels good for me to think that beyond my job, I can offer people opportunities they might not have access to without me.” (NPLM M Iran).

### Navigating (potential) conflicts within and between their roles in healthcare Language mediation (RQ2)

**Fig. 1:**
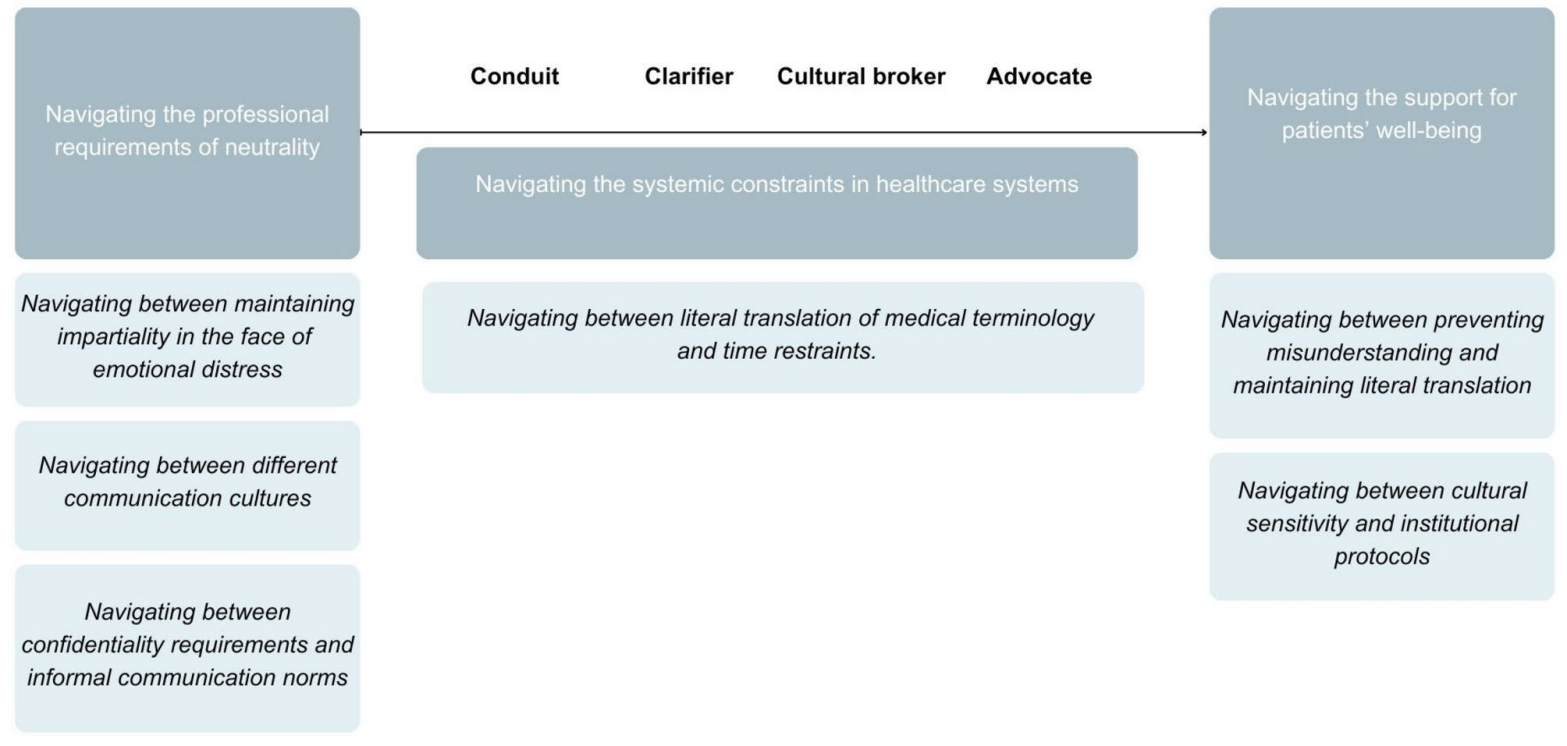
Navigating role conflicts in healthcare language mediation

This section presents the findings for RQ2, focusing on how semi-professional language mediators navigate their roles within the spectrum of conduit and advocate. Our findings highlight that mediators do not operate within rigid boundaries but adapt their strategies dynamically based on the specific needs and dynamics of each healthcare encounter (see Fig. 1).

The spectrum ranges from the neutral end, where the conduit role emphasizes neutrality and mediation with little to no alteration, to the active end, where the advocate role involves more engagement. Each sub-section describes balancing acts that mediators navigate and how they manage these fluid roles. Structural and systemic influences are also discussed in relation to how they shape these balancing acts. Table 3 gives an overview of the codes and illustrative quotes.

**Table 3:** Categories of role conflicts among semi-professional Language mediators

Main category	Subcategories	Illustrative quote
**Navigating the professional requirements of neutrality**	Navigating between confidentiality requirements and informal communication norms	“Actually, you’re only allowed to talk to the patients up there in the office because of confidentiality. When we go outside, often they will say, ‘Oh, excuse me, I have a question.’ And I say, ‘Why didn’t you ask me up there?’ These are the rules in Germany that I don’t really like. We are very hospitable people, speaking as someone from Afghanistan. Outside, people meet and talk. We’re not used to being refused [to talk]. With my knowledge of Germany or this culture, what I’ve learned here, I try to teach it and say, ‘No, we can’t discuss it here.’… That’s the balancing act we have to manage as interpreters.” (PLM M Afghanistan)
	Navigating between different communication cultures	“In our context, the doctor, I know, would say, “It’s not so bad, it’s manageable, and you can get healthy again,” and so on. They don’t tell the truth. They might try to tell the truth to the relatives, but not to the patient. They see it as protection, mostly, this is done with the elderly and children.” (NPLM F Syria)
	Navigating between maintaining impartiality in the face of emotional distress	“I believe we shouldn’t lose our humanity. That’s my opinion, we’re not supposed to [show empathy], but I do it anyway.… Sometimes I want to say something because the doctors are so fast in conveying the news. And then I just want to say from my side, ‘I’m sorry, it will be fine. Think positively.’ The important thing is that the doctor knows what I said, that’s why I always mention it. They usually don’t have a problem with it.” (PLM F Turkey)
**Navigating the support for patients’ well-being**	Navigating between preventing misunderstanding and maintaining literal translation	“I have noticed that patients with a migrant background are often somewhat less informed about their own illness and the healthcare system in general. As German doctors, or doctors trained in Germany, we are actually obliged to inform our patients about what they have, what their rights are, the best therapy, the second best, etc., so that they understand everything. Without their consent, we cannot proceed, unless it’s an emergency. This can scare them [migrants] when they hear about the risks of a treatment. They don’t understand that this is just a possibility, and think it will definitely happen to them. We have to repeat: ‘No, I have to inform you that this could happen,’ until they understand that it’s not certain.” (HCP M Palestine) “I informed the doctor that his client was feeling very insecure. So, I gave him a few warning signals. So he can think about ‘What do I do now if she jumps out of the chair?’” (NPLM F Turkey)
	Navigating between cultural sensitivity and institutional protocols	“Once there was a lady who didn’t want to be examined by a male doctor. And it was difficult to translate that because, in our culture, if you are Muslim, a strange man is not allowed to see you without a headscarf or naked. A doctor is actually allowed to do that, but at that moment, she didn’t want it, or her husband didn’t want it. Because they say, “No, absolutely no man. We want a woman.” And in that situation, it is really difficult to explain this to the doctors. But this woman would be mortified. She would die of shame, thinking the ground would open up and swallow her. It’s that kind of situation where you try to negotiate the best for the patient.” (NPLM F Turkey).
**Navigating the systemic constraints in healthcare systems**	Navigating between literal translation of medical terminology and time restraints	“Honestly, if it’s something that I think is not relevant to the conversation, I skip it because otherwise, the conversation will drag on and become even longer. But if I realize it is essential for the discussion, I will follow up, if needed, explain it.” (NPLM M Iran) “I try to convey everything. Sometimes I might do a little less because describing feelings is difficult, and clients, once they start talking, they talk endlessly.” (NPLM W Iran) “It has happened often, because as a non-native speaker, I haven’t had much to do with these terms and they are unfamiliar to me. Because it’s about sensitive topics, where things really have to be translated as accurately as possible, I then always have to ask the doctor to explain to me exactly what is meant by that.” (NPLM M Iran)

#### Navigating the professional requirements of neutrality

*Navigating between confidentiality requirements and informal communication norms.* According to many participants, although the conduit role is the expected role, with regard to the institutional guidelines of interpreter’s organizations, the performance of this role can be challenging and not desirable for various reasons. The conduit’s attribute of confidentiality was described as fitting with the German, healthcare culture, but clashed with other culture’s more informal communication styles. For instance, language mediators described encountering situations where patients or their families sought advice outside formal settings, creating a conflict between adhering to confidentiality agreements and honouring cultural expectations of openness and information sharing. To navigate this, participants explained that they often enforced confidentiality rules during formal encounters but managed informal requests with culturally sensitive explanations. They helped patients understand the importance of privacy norms while remaining approachable and respectful of their communication preferences.

*Navigating between different communication cultures* was another recurring theme among participants, requiring them to balance their role as a conduit with the need to adapt their approach to facilitate understanding between patients and healthcare providers. They described situations where a direct communication style clashed with the more indirect communication preferred by patients originating from Middle Eastern countries, e.g., when communication a severe illness. When encountering such situations, some language mediators described needing to soften direct statements from doctors to avoid causing discomfort to patients. This requires a delicate balance between remaining faithful to the original message while adapting it to be more culturally appropriate. Participants also highlighted the importance of the role of nonverbal cues which played a more important role in Middle Eastern than the German culture. Several language mediators mentioned the importance of physical connection, especially taking the patient’s hand to better convey bad news, while acknowledging their action conflicted with the expectation of maintaining a neutral stance.

*Navigating between maintaining impartiality in the face of emotional distress* also proved challenging for language mediators when faced with patients’ distress or vulnerability. Various participants described feeling a desire to show empathy and provide emotional support, particularly in situations involving bad news or difficult diagnoses. This desire to offer comfort and reassurance conflicted with their perceived obligation to remain impartial.

#### Navigating the support for patients’ well-being

*Navigating between preventing misunderstanding and maintaining literal translation* was especially pronounced among physicians who considered language mediation to “not just being about translating the language, but also about translating the background and the culture. Just translating the language is not enough, it often creates more problems than it solves” (HCP M Syria). In this, several physicians in their role as language mediators described instances where they stepped in and provided additional information as literal translations would have led to information overload or anxiety for patients who were not familiar with medical jargon, risk assessment for different treatment options, and patient involvement in medical decision-making. Likewise, several language mediators described experiences in which they provided additional explanations or advocated for clearer communication from healthcare providers.

*Navigating between cultural sensitivity and institutional protocols* was an often reported conflict. This conflict made it difficult to maintain a strictly neutral conduit role, as participants felt compelled to ensure that patients’ cultural beliefs were respected and understood by healthcare providers. The need to mediate between institutional requirements and patient preferences frequently pushed them toward more active roles, such as advocating for cultural sensitivity. According to several participants, they navigated this conflict by framing cultural preferences in terms of patient comfort and well-being, attempting to bridge the gap between patient expectations and institutional requirements. They emphasized the importance of communicating cultural nuances to healthcare providers, thereby advocating for flexibility within the established protocols. This nuanced approach allowed them to support patient needs without overtly stepping outside their role or contradicting institutional norms.

#### Navigating the systemic constraints in healthcare systems

While the previous sections focused on the fluid spectrum between neutrality and advocacy, this section addresses the underlying structural constraints of the healthcare system that further complicate the role performance of language mediators. These systemic challenges, such as time constraints and the complexity of medical terminology, often underlie and exacerbate the balancing acts mediators must navigate, forcing them to adapt their approach despite the ideals of neutrality and accuracy.

*Navigating between literal translation of medical terminology and time restraints* was a recurring theme among participants. They were acutely aware that adhering strictly to word-for-word translations could prolong appointments, which were already extended due to the language barrier. Participants described how the pressure to balance comprehensive communication with the need for efficiency often pushed them away from the ideal of literal translation. They selectively prioritized essential information to manage time effectively, acknowledging that summarizing was necessary to maintain the appointment flow. This challenge was compounded by the lack of specialized training for medical interpreting that many participants reported. With training experiences ranging widely in duration and content, from brief introductory sessions to longer certification programs, mediators often felt unprepared to handle the complexities of medical terminology and the nuanced communication required in healthcare settings. Those without a medical background, in particular, faced difficulties with unfamiliar medical terms, requiring them to seek clarifications or simplify the language for patients.

## Discussion

### Summary and discussion of study findings

In contrast to other countries like the United States, Australia, and New Zealand, where patients have a legal right to trained and accredited interpreters [45], Germany currently lacks a comprehensive legal framework mandating the provision or funding of professional language mediation services in healthcare [46, 47]. Access to interpretation in Germany remains highly fragmented: services are often arranged at the municipal level, financed variably through local government, voluntary organizations, or individual healthcare providers, and patients have no general legal entitlement to funded interpretation under the national health insurance system [7]. Although there have been political initiatives aiming to include qualified interpreting into the statutory healthcare system (SGB V), as of 2024, no binding regulation has been enacted [7]. This lack of formal infrastructure underscores the reliance on semi-professional language mediators, as investigated in this study. Against this backdrop, this study explored how semi-professional language mediators navigate the spectrum of roles, from conduit to advocate [22, 41, 48], within the context of triadic communication in healthcare encounters. We conducted qualitative interviews with *n* = 25 language mediators and physicians, examining the attributes of the conduit, clarifier, cultural broker, and advocate roles, as well as the conflicts they encounter while balancing these roles to facilitate effective communication between patients and healthcare providers.

Relating to RQ 1, our analysis revealed nuanced attributes for the four roles for language mediation within healthcare encounters. The conduit role, where the language mediator acts as a neutral channel focusing on literal translation [39, 42], was consistently highlighted as important to language mediation in healthcare. Every interview mentioned characteristics such as neutrality, confidentiality, anonymity, and accuracy to ensure impartial communication between patients and healthcare providers. Participants emphasized the importance of conveying messages without personal interpretation, maintaining privacy through confidentiality agreements, and ensuring precise communication to prevent misunderstandings. The clarifier role, focused on ensuring linguistic clarity and resolving ambiguities [39], was also frequently emphasized. Key attributes included attention to detail, communication skills, managing linguistic differences, and facilitating doctor-patient conversations. Mediators described strategies such as asking for clarification and simplified medical jargon to ensure both parties fully understood the information exchanged. Attributes of the roles of conduit and clarifier were frequently mentioned and appeared to be desired among participants.

At the same time, the cultural broker role, which involves bridging cultural gaps and ensuring that communication is culturally sensitive [37, 43], was mentioned the most in all interviews. Key characteristics included cultural mediation, providing emotional support, sensitively delivering bad news, explaining decision-making norms, building trust, and offering additional explanations. Mediators adapted their communication style to meet the cultural and emotional needs of patients, fostering better relationships with healthcare providers. The advocate role, involving standing up for the interests of the patient and ensuring their needs and rights are addressed [37, 38, 44], was mentioned the least and noted mainly among language mediators and only one doctor. Attributes associated with this role included ensuring proper medical practice, actively managing interactions, and providing continuing support beyond the interaction. Mediators intervened to prevent potential harm, clarified medical instructions, and offered additional resources and connections to support patients.

Relating to RQ 2, our findings resonate with other studies that semi-professional language mediators navigate complex and fluid roles within the spectrum of conduit and advocate [16, 49–51]. On the neutral end of the spectrum, participants reported challenges in maintaining this role, particularly when confidentiality norms or direct communication styles in the healthcare setting conflicted with patients’ cultural expectations for more open or indirect communication. To navigate these situations, mediators adhered to neutrality in formal encounters while using culturally sensitive explanations in informal contexts to respect patient preferences.

On the active end of the spectrum, the need to support patient well-being often led mediators to step beyond the constraints of the conduit role. They described instances where they intervened to prevent misunderstandings by providing additional context or advocating for clearer communication, especially when complex medical information or cultural nuances risked causing confusion or distress to patients. This more engaged approach was often described as necessary to ensure that patients fully understood their health conditions and treatment options. The differences between the sample of physicians and language mediators were subtle, with agreement that successful communication often required going beyond mere literal transmission. However, some physicians contextualized their reflections about the ambiguous responsibilities of language mediators within the framework of professional accountability. This distinction may be rooted in differing professional backgrounds as physicians can be held liable for their practice. However, notable hesitation was shared among all participants regarding the role of the advocate.

Additionally, systemic constraints, such as time pressures and the complexity of medical terminology, further complicate the mediators’ role. Participants had to prioritize conveying essential information and occasionally sought simpler explanations from healthcare providers to ensure patients could comprehend their medical situation despite the challenges posed by the healthcare environment. Overall, mediators appeared to fluidly shift between roles, aiming to balance institutional requirements with effective and culturally sensitive communication. Our insights underscore the need for discussing the theoretical and practical implications for training and supporting language mediators in managing the intricacies of triadic communication in healthcare settings.

### Theoretical implications

While frameworks of role typologies [22, 41] provide a foundational understanding, our research extends this by emphasizing the overlapping nature of these roles. Our results indicate that language mediators navigate a multitude of roles, challenging the notion of these roles as distinct and fixed entities [16, 38, 40] as they frequently shift between the conduit role and culturally adaptive forms, often aligning more closely with the roles of clarifier or cultural broker [31, 52]. However, these shifts also raise important ethical questions (e.g., which roles should be established in healthcare settings). When mediators adopt active roles, such as advocating for patients or reshaping communication, they inevitably influence the interaction beyond the mere transmission of meaning. This can blur the boundaries of their responsibilities, raising concerns about how such power is exercised and whether it might unintentionally or strategically prioritize certain perspectives over others.

The prominence of the cultural broker role, particularly in healthcare settings with diverse populations, suggests that cultural mediation warrants greater emphasis in both theoretical frameworks and training programs for language mediation [26, 53]. While this role highlights the mediator’s ability to bridge gaps and foster understanding, it also illustrates the complex power dynamics inherent in healthcare encounters. Mediators may position themselves as gatekeepers of cultural knowledge, which can shift the balance of authority in the triadic relationship. While these actions were often framed as necessary for bridging cultural or linguistic gaps, this tension reflects the dual-edged nature of mediation: it has the potential to empower marginalized patients, but it also places discretionary power in the hands of the mediator. These dynamics require careful reflection, as such practices could risk undermining the autonomy of either the patient or the provider, depending on the mediator’s interventions [17, 47]. Thus, reflections about awareness and ambiguity of roles could be paramount for language mediator training.

Furthermore, this study also highlights the recurring conflicts associated with the advocacy role, which is likely to be even more common in the use of family members as mediators in doctor-patient interactions [16, 49]. Their involvement is likely to be less neutral and associated with conflicts of interest than non-family members which can be both to the benefit or disadvantage of the patient [29, 41, 54]. It underscores the importance of engaging neutral external mediators with professional training who can carefully reflect on their roles and responsibilities [55]. As such, our study sheds light on the under-researched group of semi-professional language mediators who are increasingly employed in healthcare language mediation [40, 41, 56]. These individuals face unique challenges that necessitate clearer role definitions tailored to their specific contexts. Additionally, it is crucial to address the various conflicts that can arise during interpretation and the ethical issues arising from them. This underscores the need for a theoretical understanding of a meta-role for intercultural language mediation in healthcare [38, 53, 57].

### Practical implications

The findings highlight several practical implications for the training and support of semi-professional language mediators in healthcare settings. First, there is a clear need for targeted training programs that address the specific challenges these mediators face, such as managing cultural sensitivity, navigating emotional distress, and handling complex communication dynamics within triadic interactions in healthcare [40, 58]. Training should incorporate strategies for recognizing and managing potential role conflicts and power dynamics which can help language mediators clarify their role boundaries and use metacommunication to navigate conflicts and role expectations transparently [47, 59].

Due to the great variation in our participants’ backgrounds – from physicians with extensive medical knowledge to non-medical professional language mediators – we could not compare specific training needs regarding medical knowledge for language mediation. While training in medical terminology was considered important by our study participants, its extent and depth should be considered critically as, ultimately, language mediators need to translate complex medical information into lay language for patients. Thus, the question arises as to how much medical knowledge is necessary for effective mediation without overburdening mediators with specialized terms that might not be relevant to patient communication [60, 61]. Nonetheless, given the complex and emotionally charged nature of healthcare interactions, the BDÜ advocates for specialized training in healthcare interpreting [7]. Such training should not only cover medical terminology but also include modules on ethical considerations, confidentiality, and the emotional impact of health-related communication. In particular, training should address how mediators can responsibly manage their role when stepping into more active forms of intervention, such as advocating for patients, ensuring that such actions are transparent.

Moreover, given the rise of global migration and refugee movements, and the resulting increase in linguistically and culturally diverse healthcare settings, it is crucial to integrate trained language mediators into healthcare policy frameworks [13, 16, 17, 62]. Embedding mediators within institutional structures can help formalize their role and ensure greater accountability, while also recognizing their contributions to bridging systemic inequalities in healthcare access. This way, mediators can play a role in conveying the broader context of health systems and practices to migrant populations, which in turn would enhance their health literacy [19, 63, 64].

### Limitations

This study has several limitations that should be considered when interpreting the findings. Firstly, the research is context-specific, focusing on healthcare settings in Germany. The unique cultural and institutional dynamics of the German healthcare system may limit the applicability of the findings to other countries with different healthcare structures and cultural contexts. Secondly, the study may have a self-selection bias, as the participants were mainly engaged and reflective language mediators who volunteered to participate. This could mean that the experiences and perspectives of differently positioned mediators were not fully captured. Additionally, our participants had received a wide variety of training, ranging from language courses to medical education, which did not allow for meaningful comparisons of their training needs and competencies. Last, the fluid and overlapping nature of the roles described—conduit, clarifier, cultural broker, and advocate—posed analytical challenges. While these roles provided a valuable framework for structuring the analysis, their inherent interdependence and context-driven adaptability meant that clear distinctions between them were not always possible.

### Future research

Future research should expand on these findings by comparing the experiences of semi-professional language mediators with those of professional interpreters and family members who frequently serve as informal interpreters. Including perspectives from patients and healthcare providers would provide a more comprehensive understanding of the dynamics in language-mediated healthcare encounters. Observational studies could also offer deeper insights into the real-time challenges faced by mediators in different clinical settings. Furthermore, exploring specific health conditions or gender-specific issues, such as the additional challenges male mediators might face in gynaecological settings or when dealing with culturally sensitive topics, would help identify targeted training needs for mediators in these contexts.

## Conclusion

In conclusion, this study underscores the complex and fluid roles that semi-professional language mediators navigate within healthcare settings, balancing between the neutral conduit role and the more active advocate role. These mediators play a critical role in facilitating effective communication in culturally and linguistically diverse healthcare environments. To support their efforts, there is a pressing need for targeted training programs and policy frameworks that recognize the unique challenges they face and equip them with the skills needed to navigate the complexities of triadic communication. Addressing these needs will improve the quality of language mediation and contribute to more equitable and effective healthcare for diverse patient populations in Germany and beyond.

## Supplementary Information

Supplementary Material 1.

## Data Availability

The datasets used during the current study are available from the corresponding author on reasonable request.

## Abbreviations

F: Female
HCP: Healthcare professional, i.e., physician with language mediation experience
M: Male
NPML: Language mediator without professional healthcare experience
PML: Language mediator with professional healthcare experience
RQ: Research question

## Footnotes

1 While oral and written communication is increasingly translated by machines and technology, this paper deals exclusively with humans as a third party to transfer communication from one language to another.
