# Supplementary material for: Semi-professional language mediators in patient-provider interactions in Germany: an interview study
Source: BMC Health Serv Res. 2025 May 9;25:668. doi: 10.1186/s12913-025-12832-4 (PMC12063309; doi:10.1186/s12913-025-12832-4)
Supplement: Supplementary file 1 — Supplementary Material 1. [file 12913_2025_12832_MOESM1_ESM.pdf]

Overview of semi-structured interview guide

1) Socio-demographic data and professional experiences

- Can you share your current occupation and your path to becoming a language mediator?
- How many years of experience do you have in this field?
- Which languages do you work with? How did you acquire proficiency in them?
- Have you specialized in medical language mediation? If yes, how did this specialization come about?
- What connection, if any, do you have with specific countries or cultures?

2) Characterization of migrant groups served by the participants

- Who are the typical individuals or groups you support through language mediation in healthcare?
- How would you describe their a) understanding of and b) trust in the German healthcare system?
- Are there particular challenges or patterns that stand out in the groups you work with?

3) Medical consultations with language mediation

Roles and responsibilities

- What do you think are the most critical skills and competencies for language mediation in this context?
- How would you describe your responsibilities in a medical consultation involving language mediation?
- How would you describe or characterize your role as a language mediator? What are the attributes of this role?
- What challenges do you (often) face during medical consultations in enacting your language mediator role? How do you address these?

Triadic relationship

- How do you perceive the dynamic between the doctor, patient, and yourself during consultations?
- What challenges arise in maintaining balance between these parties?
- Can you share examples where mediation succeeded or failed, and what factors contributed to that outcome?

Competencies

- What linguistic, cultural, and interpersonal skills do you consider essential for your work?
- Do you believe medical knowledge enhances your effectiveness in this role? Why or why not?

Barriers and limitations

- What factors hinder successful mediation (e.g., trust issues, cultural misunderstandings, systemic barriers)?
- Are there situations where mediation is less effective or not feasible? Why?

4) Experiences in roles as both migrant and language mediator in the German healthcare system

- How has your migration experience shaped your work as a mediator?
- What challenges have you faced in navigating the healthcare system, personally or professionally?
- What perspectives or unique strengths do you believe you bring to this role due to your background?
- Looking forward, what changes would help improve healthcare access and communication for migrants?

Table 1: Language mediator roles, identified role-specific attributes and sample quotes

Language Mediator role	Role-specific attributes	Definition of attribute	Sample quotes
**Conduit**	Neutrality	Conveying messages without adding personal opinions, emotions, or altering the content to maintain impartiality.	"The interpreter is supposed to interpret, to convey what is being said. He should not bring in any emotions, he should not leave anything out. He should simply say what is now being said by the patient and also by the doctor." (PLM M Syria)
**Conduit**	Confidentiality	Ensuring that all information shared during the encounter	"She [the interpreter] signs a confidentiality agreement, and then I say from my side, you can trust me. I have signed that I have confidentiality.

		remains private and is not disclosed beyond the interaction.	… What you are telling me now will stay exactly here and will not end up anywhere else." (NPLM F Iran)
**Conduit**	Anonymity	Maintaining an impersonal presence, avoiding personal relationships with either party.	"We are anonymous, and they don't know who we are. We don't get a name, and they don't get a name from us either. We are a neutral, anonymous person." (NPLM F Iran<br><br>"My role as a translator must remain just as a translator, nothing more. For example, sometimes after the conversation, clients ask for my contact details. Of course, I cannot provide that. I cannot give that to them." (NPLM M Syria)
**Conduit**	Accuracy	Ensuring precise and faithful transmission of medical information, minimizing omissions and additions.	"Generally, without an interpreter, there would be no communication at all because they really don't understand a word. And medical topics are not subjects that can be conveyed with pictures or gestures. Therefore, it is really very important that everything is said precisely so that things can be communicated well." (NPLM M Iran)
**Clarifier**	Clarifying terminology	Identifying and explaining/paraphrasing complex medical terms when necessary to prevent misunderstandings.	"You can't know all the technical terms from every field. Therefore, I ask questions. If I don't understand something, I stop the conversation and say: 'Okay, what does that mean? Can you explain it a bit simpler so that I can convey it?' And if I still don't understand it, then I will also look it up digitally to understand it in general." (PLM M Afghanistan)
**Clarifier**	Managing linguistic differences	Adapting the structure or formulation of messages to	"You have to know that the Arabic language is one of the most difficult languages in the world, and there are many dialects, and people speak colloquially. There are also many cultural words that not every Arab will

		support comprehension, e.g., rephrasing or simplifying.	understand unless they come from the same culture. And that means for them that these patients actually don't understand the medical language despite a shared language on paper. You have to bring it closer to them with very simple words, with many examples." (HCP M Palestine)
**Clarifier**	Communication facilitation	Adapting the structure or formulation of messages to support comprehension, e.g., rephrasing or simplifying.	"A very, very common question that arises in many appointments is: 'Are you allergic to anything?' Especially Afghan adolescents have difficulties with this question as many of them come from small villages in Afghanistan and don't even know what an allergy means. So, I first have to explain, if you eat something, you have a negative reaction to it, and so on. So, I have to clarify the terms precisely, and they still don't understand, and then it drags on. Then the doctor thinks, what's going on here? Am I manipulating the conversation? I have to explain: 'No, I first have to explain what an allergy means, what the consequences are, what the symptoms are, and so on, because the person has never experienced it.' " (NPLM M Iran)
**Clarifier**	Managing the flow of doctor-patient-conversations	Coordinating the triadic encounter, including managing turn-taking, pace and length of speech, and when to summarize points.	"So, if the doctor, for example, talks too much, then unfortunately I can't just translate word for word, then I have to summarize." (NPLM M Iran)<br><br>"The German doctor often speaks such a long sentence. And if the patient or even I don't understand it, then he just speaks slower, but the same long sentence. I can pick out individual words from the sentence and then translate, but the patient doesn't understand, even if they know a bit of German." (HCP F Turkey)

**Cultural broker**	Cultural mediation	Bridging cultural differences by interpreting norms, behaviors, and meanings to enhance mutual understanding.	"For Turks, feelings play a more important role in relationships, so many perceive the relationship with German doctors as emotionless or very distant. And that's why it's also important that I'm there because the patient can see 'Aha, she can understand me better.' And this also helps the relationship with the doctor." (HCP F Turkey)
**Cultural broker**	'Packing' bad news	Conveying difficult medical information in a culturally sensitive way to align with patient expectations and emotional needs.	"In many cases, I find that aside from communication, it is a support for many to know that there is someone who understands me, who can relate to my culture, and I can just let go of this burden in a trusted space and enter into the conversation. Regardless of whether it really leads to a groundbreaking solution or something happens with this feeling. Just having this familiarity, I believe, gives many the energy and courage to try again. I think it is a great emotional support for many." (PLM F Iran)
**Cultural broker**	Relaying decision-making norms	Clarifying differences in medical decision-making processes to help patients engage in doctor-patient discussions.	"In Syria, the doctor decides. He says, 'we do it this way' and that's it. He rarely leaves the decision to the patients. And here it is a bit difficult because here the doctor involves the patients and they make decisions together. For people from the Middle East, that is rather unusual. I'm not suggesting that this should change, but I think it needs a bit more explanation. As a doctor myself, I can add "I know this method is good, but I want you to know what you or you have to decide too. For me, it is important that you as a patient understand the treatments and you also make the decision. Not that I don't know. I know exactly what is important." And here it plays a big role in whether the interpreter is also qualified enough to convey this. That is not always the case. This is when

			it becomes difficult because it creates uncertainty for the patients." (HCP M Syria)
**Cultural broker**	Providing additional explanations	Offering supplementary context to ensure patients understand medical procedures, expectations, and healthcare system norms.	"The doctor writes the letter and says 'everything is in the letter.' That's it for the doctor, he wrote the letter and gave the letter to the patient. But the patients don't know what to do with the letter, it has no meaning. They don't even get the idea that they have to go to the family doctor with the letter and get a prescription. Sometimes I explain it afterward when the doctor is already gone because I know that they don't know." (HCP F Turkey)
**Advocate**	Ensuring proper medical practice	Intervening to prevent miscommunication or medical decisions that could negatively impact patient safety.	"I don't just see myself as a language mediator; I cannot stand idly by when no information is being communicated to the patient or the doctor. Recently, I had a situation where a mother came to the pediatrician with a Syrian vaccination card, which was in Arabic. The pediatrician said, 'I can't do anything with this vaccination card, I will just vaccinate this child completely again.' I know you don't do that. I have a medical background, and vaccinations are not something to be done casually. I first informed the mother about this. She then asked me, 'Why does he want to vaccinate my son completely again? My son is fully vaccinated.' Then I translated this and asked the doctor, stepping out of my role as a language mediator and into my role as a medical professional, 'What about checking the [antibody] titer?' This example was my boundary because not saying 'Stop' would have been negligence on my part as a

			mediator; it would be bodily harm to re-vaccinate people completely again." (PLM W Palestine)
Advocate	Actively managing the interaction	Taking on a leadership role in the triadic encounter, e.g., by deciding when to intervene, reframe, or readdress points.	"I make sure that the doctor really addresses all the important points, in a way that is understandable. And that the patients also understand everything. For example, I repeat things myself: 'Did you understand?' Or when the doctor says she has to make an appointment here and there, I repeat it again, pretending it was the patient who asked. Because I notice during the conversation that she didn't understand it at all, which I'm not actually supposed to do. The doctors are also overwhelmed, dealing with one patient after another, emergency services, and much more. So, it is important that nothing is overlooked when it comes to health. Missing one thing could be life-threatening." (PLM W Turkey)
**Advocate**	Continuing support beyond the interaction	Continuing support beyond the interaction	"People from the Orient, particularly Persians, never say no. Even when they don't want something, they always say yes. It's similar in the Arab world. They don't allow themselves the freedom to say no or to refuse an offer, for any reason, because culturally, it would be perceived as impolite. Here, in German culture, this freedom is considered normal; if I don't want something, I don't owe anyone anything, so I simply say no. People from the Orient are different; they say yes even when they don't want to. As a language mediator, because I understand this mentality, I can guide both sides through additional questions to ensure they are both satisfied." (PLM W Iran)

"I give a bit of extra, which is not actually my job, but I do it because I have experienced it myself. Often, patients ask me if I can recommend someone or what I would do. Then I try to connect them with contacts, and it feels good for me to think that beyond my job, I can offer people opportunities they might not have access to without me." (NPLM M Iran)
